# Associations of non-high-density lipoprotein cholesterol, triglycerides and the total cholesterol/HDL-c ratio with arterial stiffness independent of low-density lipoprotein cholesterol in a Chinese population

**DOI:** 10.1038/s41440-019-0251-5

**Published:** 2019-03-27

**Authors:** Jia Wen, Yun Huang, Yao Lu, Hong Yuan

**Affiliations:** 1 Department of Cardiology, The Third Xiangya Hospital of Central South University, 410013 Changsha, China; 2 Center of Clinical Pharmacology, The Third Xiangya Hospital of Central South University, 410013 Changsha, China; 3 Pharmacy Department of Ningbo City Medical Treatment Center, Lihuili Hospital, 315040 Ningbo, China

**Keywords:** Low-density lipoprotein cholesterol, Non-high-density lipoprotein cholesterol, Triglycerides, Total cholesterol/HDL-c ratio, Brachial-ankle pulse wave velocity, Arterial stiffness, Cardiovascular disease

## Abstract

Several lipid parameters are closely associated with residual cardiovascular risk. We aimed to confirm that in a range of low-density lipoprotein cholesterol (LDL-c) levels (from <70 mg/dl to ≥160 mg/dl), other lipid parameters, such as triglyceride (TG) level, non-high-density lipoprotein cholesterol (non-HDL-c) level, and the total cholesterol (TC)/HDL-c ratio, are still related to arterial stiffness, which is a recognized marker of atherosclerosis. In this cross-sectional study, we measured brachial-ankle pulse wave velocity (baPWV), as well as clinical and biochemical indices in 16,733 Chinese adult volunteers who underwent health check-ups from January 2014 to January 2015. Arterial stiffness was defined as the upper quartile of baPWV. We applied multivariable logistic regression models to examine the associations between lipid parameters and arterial stiffness. Both men and women with high baPWV were more likely to have an atherogenic lipid phenotype. Among participants with LDL-c <70 mg/dl, participants with non-HDL-c ≥100 mg/dl had a multivariable adjusted OR for arterial stiffness of 1.66 (1.11–2.50) compared to those with non-HDL-c <100 mg/dl; participants with TG ≥150 mg/dl had an OR of 2.44 (1.61–3.71) compared to those with TG <150 mg/dl; and participants with a TC/HDL-c ratio ≥4 had an OR of 1.74 (1.15–2.65) compared to those with a TC/HDL-c ratio <4. Similar results were observed at other LDL-c levels. We found that non-HDL-c, TG, and the TC/HDL-c ratio were consistently associated with arterial stiffness in a range of LDL-c levels, even when LDL-c was below 70 mg/dl. These lipid measures are related to residual cardiovascular risk, possibly due to their detrimental effects on vascular structure.

## Introduction

Cardiovascular disease (CVD) is a public health issue worldwide [1]. Low-density lipoprotein cholesterol (LDL-c) plays a central role in the development of CVD events [2, 3]. Consequently, contemporary guidelines focus on reducing LDL-c as the primary therapeutic target [4, 5]. According to the National Cholesterol Education Program Adult Treatment Panel III Guidelines update in 2004 [4], the LDL-c goal is <100 mg/dl for high-risk patients, and a lower LDL-c goal (<70 mg/dl) is recommended for those with extremely high risk. Despite being on statin treatment, many patients with low LDL-c still experience adverse clinical outcomes; this situation is referred to as residual risk [6–8]. The predictors of residual risk include lipid-related and nonlipid factors [9, 10]. Among lipid-related factors, high-density lipoprotein cholesterol (HDL-c), triglycerides (TG), non-HDL-c, and the TC/HDL-c ratio are commonly implicated [6–8].

Arterial stiffness is a well-established marker for atherosclerosis and occurs prior to adverse cardiovascular events [11]. Brachial-ankle pulse wave velocity (baPWV), a noninvasive marker of both central and peripheral arterial stiffness, is widely used in clinical practice because of its simple methodology and good reproducibility [12]. A study on 2287 American and Japanese adults suggested a significant association between baPWV and carotid-femoral pulse wave velocity (PWV) [13]. Recent meta-analyses have established baPWV as an independent risk factor for cardiovascular events in subjects both with and without pre-existing CVD [14, 15].

Several studies have shown a strong association between lipid levels (low HDL-c, high TGs, and non-HDL-c, high TG/HDL-C and TC/HDL-C ratio, etc.) and baPWV [16–20]. However, some of those studies had a small sample size [16–18] or included only special populations [17–19]. Importantly, the associations between lipid levels and arterial stiffness in participants with low LDL-c levels have not been well studied.

The objective of the present study was to determine the impacts of TG, non-HDL-c, and the TC/HDL-c ratio on arterial stiffness as measured by baPWV in 16,733 Chinese adults. In addition, we investigated whether these associations were maintained in participants with optimal LDL-c values (<70 mg/dl).

## Patients and methods

### Study population

The participants were urban Chinese residents who had undergone an annual physical examination in The Third Xiangya Hospital of Central South University from January 2014 to January 2015. Most subjects came from Hunan Province, Jiangxi Province, and other areas located in the southern part of China. The inclusion criteria for the study included the following: (1) aged 18 years or older; (2) underwent a baPWV examination; and (3) available vital data on age, gender, body mass index (BMI), blood pressure, fasting plasma glucose (FPG), serum creatinine (Scr), and lipid profile. Initially, 16,969 participants were included. We then excluded individuals with an ankle/brachial systolic blood pressure index (ABI) < 0.95 (*n* = 206), individuals with atrial fibrillation (*n* = 44) and those undergoing regular hemodialysis (*n* = 0) [21]. Ultimately, a total of 16,733 participants (10,296 males and 6437 females) were included in the analysis.

All subjects provided informed consent before they participated in the study. The study was conducted in accordance with the Declaration of Helsinki, and the protocol was approved by the Institutional Review Board of The Third Xiangya Hospital in Changsha, China.

### Data collection

Clinical and biochemical data were obtained as previously described [22]. Briefly, baseline information on demographics, health-related habits, medical history, and current medication use was collected using a standardized questionnaire. Smoking or drinking was defined as ‘current’ (smoking or drinking in the past 6 months or quit smoking or drinking within the past 6 months), ‘former’ (had stopped smoking or drinking for more than 6 months), or ‘never’ [22]. Exercise habits were defined according to frequency per week (≤2 times/week or ≥3 times/week, with the latter indicating that the individual was physically active) [22]. Anthropometrics were measured by trained staff. Body height and weight were recorded to the nearest 0.1 cm and 0.1 kg, respectively, while participants were wearing light indoor clothing without shoes. BMI was calculated in kg/m^2^. Waist circumference (WC) was measured at the middle point between the costal margin and the iliac crest. Blood pressure (BP) was measured with the participant in the seated position based on the Joint National Committee (JNC) 7 report: [23] three readings were taken at 5-min intervals, and a mean was calculated, but if the difference between any two readings was greater than 10 mmHg, the 2 closest measurements were used. Blood samples were collected in the morning after an overnight fast of at least 8 h. Serum FPG, TG, TC, LDL-c, HDL-c, and Scr levels were determined using an autoanalyzer (Hitachi 7600–110; Hitachi, Tokyo, Japan). Non-HDL-c was calculated as TC minus HDL-c.

We measured baPWV with a noninvasive atherosclerosis measurement system (VP-2000; Colin Co Ltd, Komaki, Japan). All the subjects were asked to refrain from smoking or alcohol before the tests. Measurements were taken in a room at constant temperature after the individuals had rested in the supine position for 10 min. Four pneumatic cuffs were attached to bilateral arms and ankles to measure pulse waves [24]. The baPWV was automatically calculated as follows: baPWV = (L_a_−L_b_)/T_ba_ (L_a_ is the distance from the heart to the ankle, L_b_ is the distance from the heart to the brachium, and T_ba_ is the time difference between the initial increase in the brachial waveform and that in the ankle waveform) [24]. ABI (the ratio of SBP in the leg to that in the arm on the same side) and heart rate were also automatically recorded.

### Definitions of hypertension, diabetes, and dyslipidemia

Hypertension was defined as systolic BP (SBP) ≥140 mmHg, diastolic BP (DBP) ≥90 mmHg or the use of antihypertensive medication. Diabetes was defined as FPG ≥ 126 mg/dl or the use of insulin or oral hypoglycemic medication. Low HDL-c was defined as <40 mg/dl in men and <50 mg/dl in women. Dyslipidemia was defined as LDL-c ≥ 140 mg/dl, TG ≥ 150 mg/dl, low HDL-c or current use of antidyslipidemic medication. High baPWV was defined as the upper quartile of baPWV [25].

### Statistical analysis

Continuous data are expressed as the mean ± standard deviation or median with interquartile range, and categorical data are expressed as proportions. Continuous data for participants with high baPWV and those with low baPWV were compared with the *t*-test or the Mann–Whitney *U* test, and categorical data were analyzed by the chi-square test. The multivariable logistic regression model was utilized to evaluate the association of high baPWV with predefined cut-off values of LDL-c (<70 mg/dl, 70–99.9 mg/dl, 100–129.9 mg/dl, ≥130 mg/dl), non-HDL-c (<100 mg/dl, 100–129.9 mg/dl, 130–159.9 mg/dl, ≥160 mg/dl), TGs (<150 mg/dl, 150–199.9 mg/dl, 200–249.9 mg/dl, ≥ 250 mg/dl), and the TC/HDL-c ratio (<4.00, 4.00 to 4.99, 5.00 to 5.99, ≥6.00) based on current guidelines and studies [4, 8]. Because of the skewed distribution, TG levels were log-transformed. Each lipid parameter was then examined as a continuous variable (per 1-SD increment) in the abovementioned model. We also calculated the odds ratios (ORs) for high baPWV with elevated non-HDL-c (30 mg/dl higher than the maximal LDL-c level), TGs (≥150 mg/dl), or TC/HDL-c ratio (≥4.00) across different levels of LDL-c (<70 mg/dl, 70–99.9 mg/dl, 100–129.9 mg/dl, and ≥130 mg/dl). All the analyses were adjusted for variables associated with PWV, including age, sex (total), smoking and drinking status, physical activity, BMI, HR, FPG, SBP, pulse pressure, Cr, low HDL-c, and medications for diabetes, hypertension, or dyslipidemia [21, 26]. The variance inflation factor (VIF) was used to detect collinearity, with a VIF ≥ 10 indicating a collinearity problem. Statistical analyses were performed with SPSS 22.0 (SPSS Inc., Chicago, IL). We corrected for multiple comparisons by adjusting for the number of independent tests using the Li and Ji method [27]. Thus, a *P* value less than 0.025 (0.05/2) was considered to indicate statistical significance.

## Results

The mean age of the entire cohort was 48.4 years, and 62% of the participants were male. Among the male study participants, 26.5% were in the high baPWV group, and among the female study participants, 22.7% were in the high baPWV group. The general characteristics of the high baPWV and low baPWV groups stratified by gender are described in Table 1. Among both genders, the high baPWV group was older and more frequently had a history of hypertension, diabetes, and dyslipidemia than the low baPWV group. Antihypertensive, hypoglycemic, and lipid-lowering agents were more frequently used in the high baPWV group. Higher BMI, WC, HR, and BP and FPG, TG, TC and non-HDL-c levels were also detected in the high baPWV group. However, LDL-c and the TC/HDL-c ratio were significantly different between women with and without high baPWV but not between men with and without high baPWV.

**Table 1:** Characteristics of study subjects stratified by brachial-ankle pulse wave velocity and gender

	Men		Women	
	Low baPWV	High baPWV	Low baPWV	High baPWV
Number	7572	2724	4978	1459
Age (years)	45.3 ± 9.7	57.3 ± 11.9^c^	45.2 ± 9.9	59.3 ± 9.1^c^
Current smoking (%)	57.4	48.9^c^	5.2	3.5^a^
Current drinking, (%)	70.9	58.8^c^	27.4	15.6^c^
Physical activity (%)	37.6	52.6^c^	40.2	51.2^c^
BMI (kg/m^2^)	25.2 ± 3.1	25.4 ± 3.1^a^	23.0 ± 3.1	24.6 ± 3.0^c^
WC (cm)	87.1 ± 8.6	88.2 ± 8.5^c^	76.9 ± 8.3	82.7 ± 8.4^c^
HR (b/min)	70.4 ± 10.5	75.6 ± 12.3^c^	71.1 ± 10.1	74.6 ± 11.9^c^
SBP (mmHg)	122.2 ± 12.3	138.7 ± 6.1^c^	115.8 ± 13.6	139.8 ± 17.3^c^
DBP (mmHg)	78.0 ± 10.0	86.2 ± 12.1^c^	71.8 ± 9.6	81.8 ± 11.6^c^
Pulse pressure (mmHg)	44.2 ± 8.3	52.6 ± 12.6^c^	44.0 ± 9.0	58.1 ± 13.4^c^
Hypertension (%)	18.4	61.4^c^	8.9	60.1^c^
Antihypertensive drugs (%)	6.1	25.4^c^	3.8	28.8^c^
FPG (mg/dl)	96.6 ± 20.8	108.8 ± 37.3^c^	92.1 ± 13.1	104.9 ± 31.2^c^
Diabetes (%)	4.7	13.4^c^	1.5	10.3^c^
Antidiabetic drugs (%)	2.1	7.6^c^	0.8	6.0^c^
TG, mg/dl	140.9(97.5–208.3)	145.8(100.2–221.4)^b^	90.4(67.4–127.6)	127.6(93.9–176.4)^c^
TC (mg/dl)	202.5 ± 38.1	206.3 ± 44.5^c^	198.0 ± 37.4	215.1 ± 39.6^c^
LDL-c (mg/dl)	111.9 ± 32.7	110.5 ± 34.6	107.4 ± 31.9	120.0 ± 34.1^c^
HDL-c (mg/dl)	55.4 ± 13.4	56.9 ± 14.9^c^	69.1 ± 15.4	65.5 ± 15.2^c^
Non-HDL-c (mg/dl)	147.1 ± 38.2	149.5 ± 44.3^a^	128.9 ± 36.9	149.6 ± 38.3^c^
TC/HDL-c ratio	3.84 ± 1.02	3.83 ± 1.09	2.98 ± 0.79	3.41 ± 0.83^c^
Dyslipidaemia (%)	56.7	59.9 ^b^	28.5	53.1^c^
Lipid-lowering drugs (%)	1.0	2.7 ^c^	0.9	2.3^c^
Serum creatinine (mg/dl)	0.87 ± 0.14	0.90 ± 0.26^c^	0.61 ± 0.10	0.66 ± 0.21^c^
eGFR, mL/min/1.73 m^2^	108.9 ± 21.4	103.4 ± 24.9^c^	132.9 ± 28.3	118.6 ± 30.1^c^
baPWV (cm/s)	1299.6 ± 129.9	1777.7 ± 263.9^c^	1239.4 ± 150.7	1794.7 ± 266.3^c^

Values are presented as the mean ± standard deviation (median with interquartile range) or proportion

*BMI* body mass index, *WC* waist circumference, *HR* heart rate, *SBP* systolic blood pressure, *DBP* diastolic blood pressure, *FPG* fasting plasma glucose, *TG* triglyceride, *TC* total cholesterol, *LDL-c* low-density lipoprotein cholesterol, *HDL-c* high-density lipoprotein cholesterol, *eGFR* estimated glomerular filtration rate, *baPWV* brachial-ankle pulse wave velocity

^a^*P* < 0.05, ^b^*P* < 0.01, ^c^*P* < 0.001

Table 2 shows the adjusted ORs for the prevalence of high baPWV according to LDL-c, non-HDL-c, and TG levels and the TC/HDL-c ratio. No collinearity was observed between variables. Compared to participants with non-HDL-c < 100 mg/dl, the multivariable adjusted ORs ranged from 1.25 (95% CI, 1.04–1.51) among participants with non-HDL-c of 100–129.9 mg/dl to 1.46 (95% CI, 1.22–1.75) among participants with non-HDL-c ≥160 mg/dl (*P* < 0.001 for trend). Similarly, elevated TGs and the TC/HDL-c ratio were associated with a greater prevalence of high baPWV. No significant association was detected between high baPWV and LDL-c, and there were no significant interactions between sex and lipid categories.

**Table 2:** ORs for high baPWV stratified by LDL-c, non-HDL-c, and TG levels and the TC/HDL-c ratio

LDL-c (mg/dl)	<70	70–99.9	100–129.9	≥130	*P* for trend
Total (OR)	1.00	0.92(0.76–1.11)	0.91(0.76–1.09)	0.98(0.81–1.18)	0.701
Men (OR)	1.00	0.86(0.69–1.06)	0.81(0.66–1.00)	0.87(0.70–1.08)	0.427
Women (OR)	1.00	1.15(0.78–1.70)	1.17(0.80–1.70)	1.18(0.80–1.73)	0.556

Non-HDL-c (mg/dl)	<100	100–129.9	130–159.9	≥160	
Total (OR)	1.00	1.25(1.04–1.51)	1.18(0.99–1.42)	1.46(1.22–1.75)	<0.001
Men (OR)	1.00	1.24(0.98–1.58)	1.12(0.89–1.41)	1.36(1.08–1.71)	0.015
Women (OR)	1.00	1.17(0.85–1.62)	1.13(0.83–1.54)	1.36(0.99–1.86)	0.055

TGs (mg/dl)	<150	150–199.9	200–249.9	≥250	
Total (OR)	1.00	1.09(0.96–1.25)	1.33(1.12–1.58)	1.48(1.28–1.72)	<0.001
Men (OR)	1.00	1.06(0.90–1.25)	1.30(1.07–1.59)	1.51(1.27–1.78)	<0.001
Women (OR)	1.00	1.06(0.83–1.34)	1.27(0.90–1.80)	1.19(0.82–1.69)	0.209

TC/HDL-c	<4.00	4.00–4.99	5.00–5.99	≥6.00	
Total (OR)	1.00	1.19(1.06–1.34)	1.27(1.05–1.55)	1.56(1.09–2.21)	<0.001
Men (OR)	1.00	1.11(0.97–1.28)	1.22(0.99–1.50)	1.50(1.04–2.18)	0.009
Women (OR)	1.00	1.37(1.07–1.76)	1.35(0.79–2.30)	1.62(0.50–5.26)	0.013

The values are presented as the odds ratio (OR) (95% confidence interval); ORs were obtained after adjusting for age, sex (total), smoking and drinking status, physical activity, BMI, HR, FPG, SBP, pulse pressure, Cr, low HDL-c, and medications for diabetes, hypertension, and dyslipidaemia

*OR* odds ratio; other abbreviations are as listed in Table 1

Table 3 presents the adjusted ORs for the prevalence of high baPWV associated with a 1-SD increase in lipid profile. After adjusting for all the confounding factors, the ORs for high baPWV per 1-SD increase in lipid profile were 1.12 (95% CI, 1.06–1.17) for non-HDL-c, 1.22 (95% CI, 1.16–1.29) for TG, and 1.15 (95% CI, 1.09–1.22) for the TC/HDL-c ratio. Again, LDL-c was not associated with high baPWV. We found a significant interaction between sex and LDL-c in the prediction of high baPWV risk (*P* = 0.025), but there were no other sex-lipid interactions.

**Table 3:** ORs for high baPWV per 1-SD increase in LDL-c, non-HDL-c, and TG levels and the TC/HDL-c ratio

	SD	OR (95% CI)	*P* value
LDL-c			
Total	33.1	1.01(0.96–1.06)	0.653
Men	33.3	0.97(0.91–1.03)	0.275
Women	32.8	1.05(0.96–1.15)	0.278
Non-HDL-c			
Total	39.9	1.12 (1.06–1.17)	<0.001
Men	39.1	1.10 (1.04–1.17)	0.002
Women	38.2	1.08 (0.99–1.19)	0.087
TGs^a^			
Total	149.1	1.22 (1.16–1.29)	<0.001
Men	172.4	1.22 (1.14–1.30)	<0.001
Women	85.8	1.16 (1.03–1.30)	0.012
TC/HDL-c			
Total	1.02	1.15 (1.09–1.22)	<0.001
Men	1.03	1.12 (1.05–1.20)	0.001
Women	0.82	1.17 (1.04–1.32)	0.009

The values are presented as the odds ratio (OR) (95% confidence interval); ORs were obtained after adjusting for age, sex (total), smoking and drinking status, physical activity, BMI, HR, FPG, SBP, pulse pressure, Cr, low HDL-c, and medications for diabetes, hypertension, and dyslipidaemia

*SD* standard deviation; other abbreviations are as listed in Table 1

^a^The variable is presented as the original value after analysis using the log-transformed value

Table 4 shows the association between non-HDL-c, TGs, or the TC/HDL-c ratio and high baPWV across a range of LDL-c values from <70 mg/dl to ≥130 mg/dl. Even among individuals with LDL-c <70 mg/dl, elevated non-HDL-c, TGs and TC/HDL-c ratio were associated with high baPWV. Among participants with an LDL-c of 100–129.9 mg/dl, non-HDL-c and TGs as categorical variables were not independent risk factors for high baPWV, but non-HDL-c and TGs as continuous variables revealed a significant association.

**Table 4:** ORs for high baPWV, stratified by non-HDL-c and TG levels and the TC/HDL-c ratio, among participants classified based on LDL-c levels

	Non-HDL-c < 100	Non-HDL-c ≥ 100	TG < 150	TG ≥ 150	TC/HDL-c < 4	TC/HDL-c ≥ 4
LDL-c < 70	1.00	1.66 (1.11–2.50)	1.00	2.44 (1.61–3.71)	1.00	1.74 (1.15–2.65)
	Non-HDL-c < 130	Non-HDL-c ≥ 130				
LDL-c 70–99.9	1.00	1.46 (1.15–1.85)	1.00	1.42 (1.12–1.80)	1.00	1.39 (1.07–1.81)
	Non-HDL-c < 160	Non-HDL-c ≥ 160				
LDL-c 100–129.9	1.00	1.20 (0.96–1.50)	1.00	1.19 (0.95–1.50)	1.00	1.06 (0.87–1.29)
	Non-HDL-c < 160	Non-HDL-c ≥ 160				
LDL-c ≥ 130	1.00	1.41 (1.10–1.81)	1.00	1.40 (1.09–1.78)	1.00	1.25 (1.04–1.51)
	Per 1-SD increase in non-HDL-c		Per 1-SD increase in TGs^a^		Per 1-SD increase in TC/HDL-c	
LDL-c < 70	1.17 (1.02–1.36)		1.29 (1.13–1.47)		1.21 (1.05–1.41)	
LDL-c 70–99.9	1.33 (1.17–1.52)		1.15 (1.04–1.27)		1.07 (0.95–1.20)	
LDL-c 100–129.9	1.16 (1.00–1.34)		1.16 (1.04–1.28)		1.07 (0.96–1.20)	
LDL-c ≥ 130	1.13 (1.00–1.28)		1.25 (1.10–1.42)		1.18 (1.06–1.32)	

The values are presented as the odds ratio (OR) (95% confidence interval); ORs were obtained after adjustments for age, sex, smoking and drinking status, physical activity, BMI, HR, FPG, SBP, pulse pressure, Cr, low HDL-c, and medications for diabetes, hypertension, and dyslipidaemia

*OR* odds ratio; other abbreviations are as listed in Table 1

^a^The variable is presented as the original value after analysis using the log-transformed value

## Discussion

In this large population-based study, we observed that non-HDL-c, TG, and the TC/HDL-c ratio, but not LDL-c, were consistently and positively associated with arterial stiffness, as defined by high baPWV, independent of CVD risk factors. In addition, positive associations were consistently observed for non-HDL-c, TGs and the TC/HDL-c ratio within every LDL-c level investigated, including LDL-c <70 mg/dl. TGs may be more predictive of arterial stiffness than non-HDL-c and the TC/HDL-c ratio.

Our observation shows that non-HDL-c, TG, and the TC/HDL-c ratio were associated with arterial stiffness is consistent with most previous studies, although different indices were adopted [16–20]. Non-HDL-c was calculated as TC minus HDL-c and thus included all atherogenic lipoproteins (VLDL, intermediate-density lipoprotein cholesterol (IDL-c), LDL-c, and lipoprotein(a)) [5]. Non-HDL-c was identified as a good predictor of subclinical atherosclerosis in a Dutch cohort including 1517 individuals based on the detection of a lower ABI, a higher augmentation index, thicker plaques, increased PWV, and increased intima-media thickness [16]. Similarity, Ma et al. [17]. determined that non-HDL-c was independently correlated with carotid intima-media thicknesses in normotensive and normoglycemic females. Elevated nonfasting serum TGs indicates elevated lipoprotein remnants (VLDL, IDL-c, and chylomicrons remnants), which can penetrate the vascular endothelium and lead to atherosclerosis [28]. Two recent studies involving Chinese adults who underwent health examinations demonstrated that elevated TG levels were associated with baPWV [18, 20]. Another study of 2351 Caucasian adults revealed a positive association between TG levels and baPWV in males [19]. The TC/HDL-c ratio has been suggested as a useful cumulative index of the atherogenic lipid phenotype and was found to be associated with insulin resistance in the Quebec Cardiovascular Study [29]. Moreover, data from two prospective cohorts [30, 31] involving 14,916 initially healthy US males and 27,935 females confirmed that the TC/HDL ratio was significantly associated with peripheral arterial disease incidence. We are the first to report the association between the TC/HDL ratio and PWV. Our study did not demonstrate a relationship between LDL-c and high baPWV, which is in line with previous findings [18, 32]. We suspect that the influence of LDL-c was hidden by other, stronger risk factors, such as age, hypertension, and diabetes. Interestingly, Brinkley et al. [33] showed that elderly participants with elevated oxidized LDL-c were 30 to 55% more likely to have high aortic PWV.

LDL-c is the primary target of therapy according to current guidelines; [4, 5] however, whether any of the aforementioned relationships remain when the LDL-c is lower continues to be an unsolved but important issue. Meanwhile, other on-treatment lipid levels are related to a residual risk of CVD among a subset of subjects with low LDL-c [6–8]. In a post hoc analysis of the TNT (Treating to New Targets) and IDEAL (Incremental Decrease in End Points through Aggressive Lipid Lowering) trials [6], the hazard ratios for future cardiovascular events (per 1-SD increase) were 1.15 (1.05–1.25) for non-HDL-c, 1.15 (1.05–1.25) for apolipoprotein B, 1.22 (1.14–1.30) for the TC/HDL-c ratio, and 1.31 (1.21–1.41) for the apolipoprotein B/A-1 ratio among patients with LDL-c under 100 mg/dl; however, TGs were not evaluated. In the EPIC (European Prospective Investigation into Cancer and Nutrition)-Norfolk study [7], non-HDL-C, TGs, and the TG/HDL-c ratio were all strongly associated with future coronary heart disease (CHD), with hazard ratios of 1.84 (1.12–3.04), 1.63 (1.02–2.59), and 2.19 (1.22–3.93), respectively, among individuals with LDL-c below 100 mg/dl. In the subanalysis of participants with LDL-c less than 100 mg/dl in the JUPITER (Justification for the Use of Statins in Prevention: An Intervention Trial Evaluating Rosuvastatin) study [8], non-HDL-c was valuable in predicting CVD incidence, and the TC/HDL-c ratio was borderline significant, whereas TGs were not associated with CVD. To the best of our knowledge, the present study is the first to report significant positive associations of non-HDL-c, TGs, and the TC/HDL-c ratio with arterial stiffness irrespective of LDL-c level, especially among participants with naturally very low LDL-c (<70 mg/dl). Our research adds novel evidence that these lipid parameters are closely associated with high residual cardiovascular risk, possibly because of additional deleterious effects on arterial stiffness.

Previous evidence generally supports a causal relationship between lipids and vascular stiffness. Several clinical trials have demonstrated that lipid-lowering therapy improves baPWV [34–37]. A recent meta-analysis by Upala et al. [38] involving 303 participants also reported that statin therapy was associated with lower aortic PWV. However, it should be noted that limited numbers of participants have been included in these studies. In addition, several lifestyle changes, including weight loss, physical exercise, salt reduction, and cessation of smoking or drinking, have beneficial effects on vascular stiffness [11].

Our study has certain limitations. First, the cohort was voluntary, and individuals already under treatment for CVD may not have participated. Therefore, the study may be biased by the healthy worker effect [39]. Second, our cohort consisted of mostly urban participants who underwent health examinations. These subjects were relatively younger, with a mean age of 48.4 ± 11.5 years, and thus might have been more likely to be concerned about their health than the general population. For example, the prevalence of diabetes mellitus and obesity in this population was lower than that in the general Chinese population (5.6% vs. 11.6% and 4.7% vs. 12.0%, respectively) [40]. Moreover, HDL-c was higher in these participants than in the general population (60.6 ± 15.7 mg/dl vs. 51.7 ± 11.4 mg/dl) [41], and fewer were on statin treatment (2.6% vs. 6.84%) [42]. Therefore, our findings may be generalized to only relatively healthy and young populations. Finally, due to the cross-sectional study design, it was difficult to infer causality between dyslipidemia and arterial stiffness in our study. Therefore, prospective studies are required to examine the predictive value of the lipid profile for vascular risk in the general population.

In conclusion, our study extends the results of previous studies by demonstrating the associations of non-HDL-c, TG, and the TC/HDL-c ratio with arterial stiffness over a range of LDL-c concentrations, even those that are optimal (<70 mg/dl), among 16,733 relatively healthy individuals. These findings suggest a potential mechanism underlying the residual cardiovascular risk observed in patients with low LDL-c.
